# Influence of Au, Pt, and C Seed Layers on Lithium Nucleation Dynamics for Anode-Free Solid-State Batteries

**DOI:** 10.1021/acsami.3c14693

**Published:** 2023-12-21

**Authors:** André Müller, Luis Paravicini, Jȩdrzej Morzy, Maximilian Krause, Joel Casella, Nicolas Osenciat, Moritz H. Futscher, Yaroslav E. Romanyuk

**Affiliations:** Laboratory for Thin Films and Photovoltaics, Empa—Swiss Federal Laboratories for Materials Science and Technology, Überlandstrasse 129, Dübendorf CH-8600, Switzerland

**Keywords:** seed layers, lithium plating/stripping, anode-free, solid-state battery

## Abstract

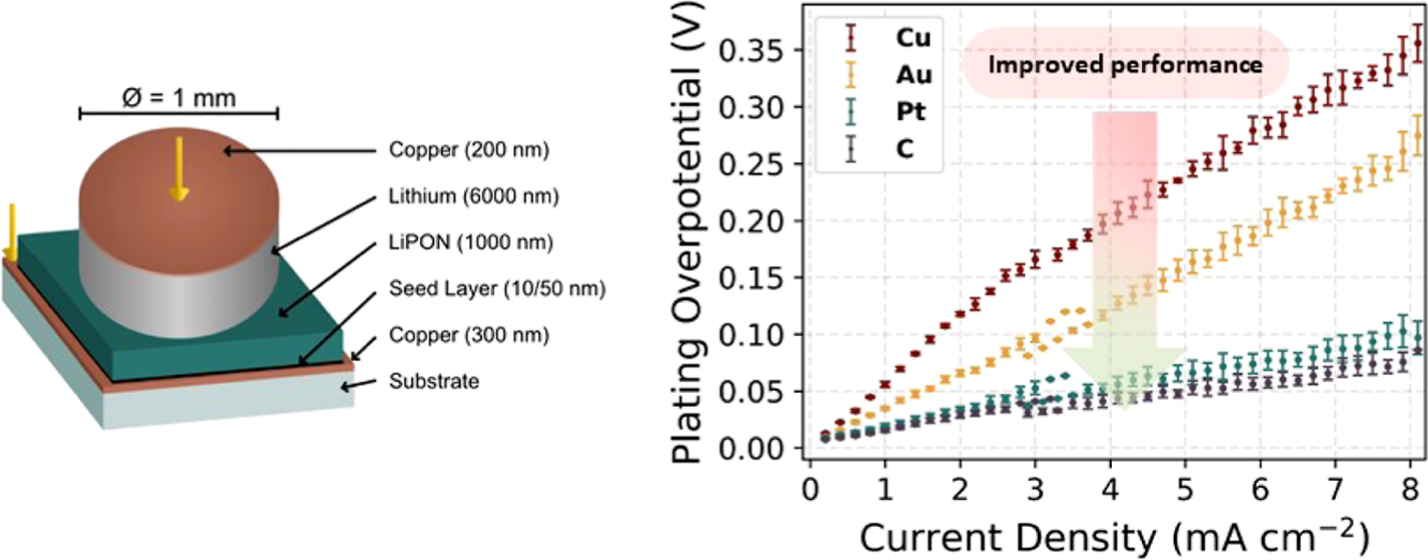

In the concept of anode-free lithium-ion batteries, cells are manufactured with a bare anode current collector where the lithium metal anode is electrochemically formed from the lithium-containing cathode during the first charge cycle. While this concept has many attractive aspects from a manufacturing and energy density standpoint, stable plating and stripping remain challenging. We have investigated gold, platinum, and amorphous carbon as seed layers placed between the copper current collector and the lithium phosphorus oxynitride thin-film solid electrolyte. These layers guide lithium nucleation and improve the plating and stripping dynamics. All seed layers facilitate reversible lithium plating and stripping even at high current densities up to 8 mA cm^–2^. Of particular note is the amorphous carbon seed layer, which allowed a significant reduction in plating potential from 300 mV to as low as 50 mV. These results underscore the critical role of seed layers in improving the efficiency of anode-free solid-state batteries and open the door to simplified manufacturing of anode-free battery designs.

## 1 Introduction

Solid-state batteries are considered the next generation of battery technology, offering advantages in safety and energy density.^1−5^ The introduction of a solid, ionically conductive lithium-ion electrolyte in place of the organic liquid electrolyte traditionally used in lithium-ion batteries promises longer life and improved safety by eliminating flammable components. This technology also paves the way for replacing traditional graphite anodes with metallic lithium, resulting in 40–50% higher energy density.^6−10^ The introduction of metallic lithium presents challenges such as high reactivity, unstable interfaces, and lithium dendrite growth due to nonuniform plating and stripping.^11^ These complications can lead to current focusing, dendrite formation during battery cycling, and potentially dangerous short circuits. Furthermore, lithium metal is highly reactive, and integration into a battery is only possible under an inert atmosphere, suggesting that the use of metallic lithium films in solid-state batteries may not be practical.^12^

One potential strategy to overcome manufacturing challenges and further increase energy density is to move away from lithium foils to anode-free solid-state batteries (AFSSBs) or a “zero lithium excess” manufacturing process.^13^ Here, the battery is manufactured in the discharged state with a lithium-containing cathode and a bare anode-side current collector (CC).^14−17^ This concept not only increases the energy density by reducing the battery volume and weight but also reduces the handling and manufacturing complexity. The lithium metal anode is then formed electrochemically during the first charge cycle by electroplating lithium present in the cathode. Therefore, mechanisms that control the nucleation and growth of lithium metal are crucial to the success of AFSSBs.^18^ While the concept of anode-free batteries had been previously demonstrated in traditional liquid systems,^17,19−22^ its implementation in solid-state systems lagged.

Early research on AFSSBs began with a study by Neudecker et al.^23^ in 2000. This work involved the fabrication of an anode-free thin film battery with a copper current collector, a lithium phosphorus oxynitride (LiPON) electrolyte, and a lithium cobalt oxide cathode using magnetron sputtering. The battery retained 80% of its original capacity after 1000 cycles. Later research shifted the focus to an anode-free battery with a seed layer. A seed layer is a comparable thin layer deposited between the anodic current collector and the solid electrolyte. It provides nucleation sites for lithium metal growth and can improve battery performance and stability.^13^ This direction was notably advanced by Lee et al.^24^ at Samsung in 2020. Their research demonstrated an AFSSB with a silver–carbon nanocomposite layer and a sulfide electrolyte, achieving more than 1000 cycles and an energy density of more than 900 W h L^–1^. The study of Feng et al.^25^ demonstrated the effectiveness of carbon seed layers in improving the air stability of LLZO when deposited on a garnet-based electrolyte, thereby reducing the area-specific resistance of the Li/LLZO interface. Building on this, Futscher et al.^26^ explored the use of amorphous carbon seed layers. These layers facilitated uniform lithium plating, effectively prevented dendrite formation, and increased the critical current density to 8 mA cm^–2^.

Besides carbon interlayers and mixtures of carbon composites, noble metals such as platinum and gold seed layers have also been explored. Studies on platinum^27,28^ revealed the effects of lithium plating and stripping reactions with platinum current collectors on LiPON, increasing the lithium nucleation number density compared to copper CC. Microscopic observations provided insights into the interactions between platinum and lithium. Recent studies of gold seed layers^29−34^ have shown their role in improving the efficiency and lifetime of AFSSBs. The work of Krauskopf et al.^32^ has investigated the effects of morphological instability of lithium metal anodes in the presence of gold seed layers. They have shown that the use of a lithium-alloying gold layer delays the penetration of lithium metal into the garnet electrolyte and penetration occurs only after the alloy phases are fully formed. This line of research was further explored by Kim et al.,^33^ who demonstrated effective regulation of lithium distribution on LLZO by modifying the surface with an interlayer. They proposed that the seed interlayer serves two main functions during battery operation: it acts as a dynamic buffer for the redistribution of lithium and as a matrix layer for facile lithium precipitation.

This work aims to compare the impact of different seed layers — gold, platinum, and amorphous carbon — on lithium plating and stripping in a thin-film configuration. The seed layers are placed between the bare copper CC—since the early day being used as conventional current collectors on the anode side^35^—and the LiPON solid electrolyte. The resulting configurations were tested in half-cell structures, and the evolution of the overpotential and the relationship between lithium plating/stripping, nucleation kinetics, and alloying properties of each seed layer were analyzed. By comparing different seed layers, amorphous carbon was found to be a cost-effective alternative to precious metals, reducing the rise in overpotential by up to 70%.

## 2 Results and Discussion

**Figure 1:**
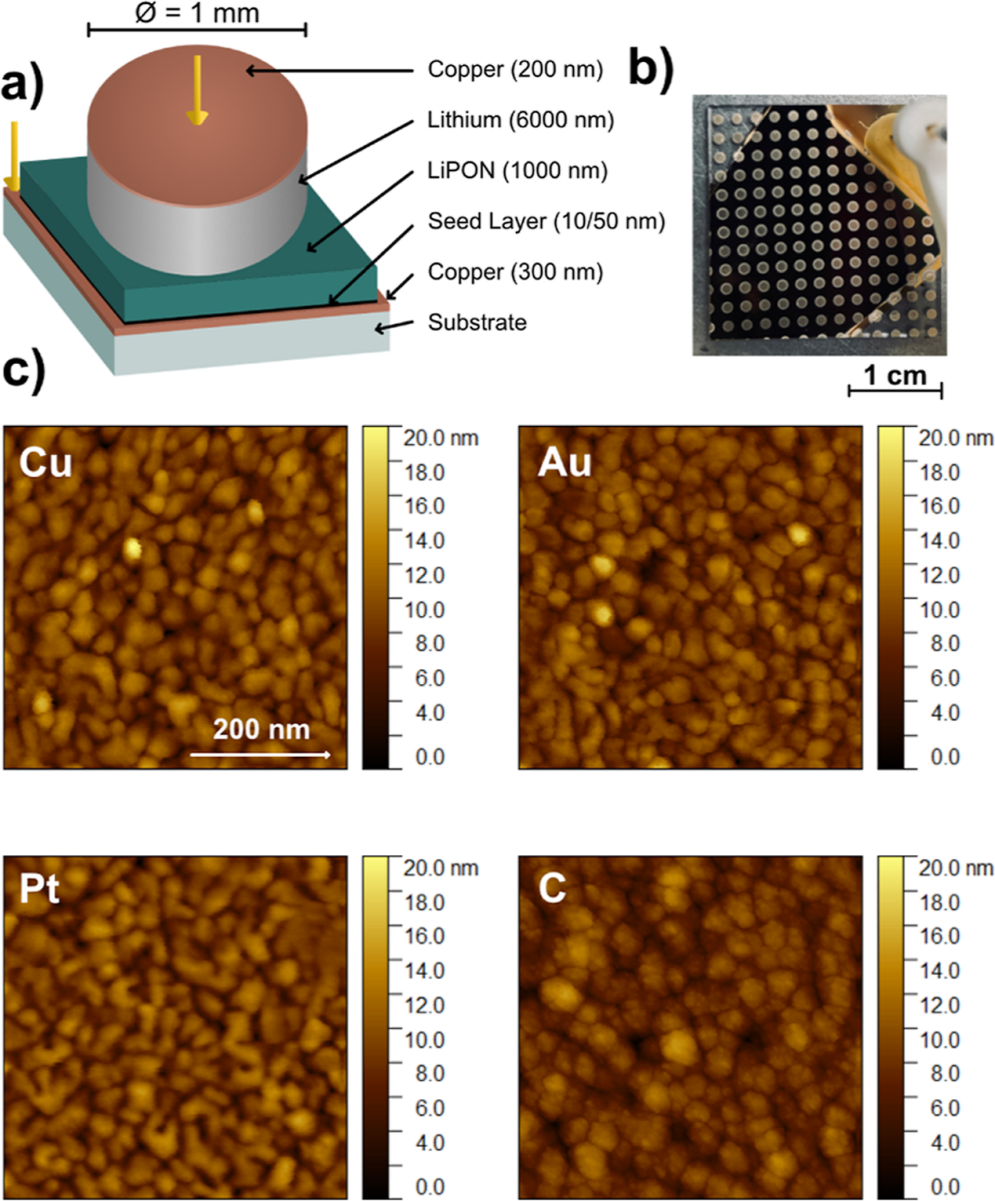
(a) Schematic illustration of the device configuration: Cu/Seed layer/LiPON/Li/Cu. (b) Photograph showing separate lithium reservoirs (1 mm diameter) for distinct cells. (c) AFM micrographs of bare copper CC, gold, platinum, and amorphous carbon seed layers. The scale bar is the same for all micrographs.

We fabricated thin-film stacks to study different seed layer materials in the following architecture: Cu/seed layer/LiPON/Li/Cu, as shown in Figure 1a, and compared with the reference architecture: Cu/LiPON/Li/Cu. The use of shadow masks allowed us to evaporate individual areas (1 mm diameter dots) of the lithium reservoir as separate cells (Figure 1b). The LiPON solid electrolyte was chosen due to its successful track record in facilitating reversible cycling of lithium in AFSSBs, especially when combined with copper as a current collector.^23^ The amorphous nature of LiPON isolates the surface morphology and chemistry from other potential obstacles, such as the presence of grain boundaries—a notable advantage over crystalline electrolytes such as LLZO.^36^ In addition, LiPON’s ability to form a thin yet stable passivation layer with the lithium metal helps reduce lithium loss during subsequent cycling.^37,38^

Gold, platinum, and amorphous carbon were chosen as the seed layer materials. A 10 nm thick layer of gold was deposited by thermal evaporation. Platinum and carbon layers were deposited by RF magnetron sputtering to achieve thicknesses of 10 nm for platinum and 50 nm for carbon. Initial tests showed that the 10 nm gold layer in our thin film battery showed superior cycling performance compared to thicker layers (100 nm gold layer, Figures S1 and S2). The comparatively poor performance of thicker films compared with thinner films is due to the longer lithium diffusion length. This results in greater internal stress during lithium insertion/extraction.^39^ Amorphous carbon, on the other hand, was previously shown to perform best at a thickness of 50 nm.^26^

The atomic force microscopy (AFM) images shown in Figure 1c reveal surface morphologies that are comparable for all seed layers. All materials exhibit a smooth texture with an RMS roughness of 2.2 nm ± 0.1 nm. There are no significant morphological differences between the different seed layers. For a complete overview, see Table S1 in the ESI. Therefore, it is unlikely that the morphological characteristics of the seed layers have an influence on the lithium plating and stripping processes. As a result, observed disparities can be attributed to differences in electrochemical processes and physicochemical properties such as interfacial energies, alloying energies, and so forth.

Experiments involving the plating and stripping of a dense lithium metal layer were conducted. In our study, the terms “plating and stripping” in the context of thin film anode-free half cells refer to the process of galvanostatic charging and discharging. This involves the application of a constant current, which is essential to manage the plating and stripping of lithium. We set constant current conditions for a certain duration to obtain a lithium layer of 250 nm or 1 μm depending on the experiment. In addition, we set potential limits to 1.5 V vs Li/Li^+^ to avoid excessive degradation of the LiPON solid-electrolyte layer.

**Figure 2:**
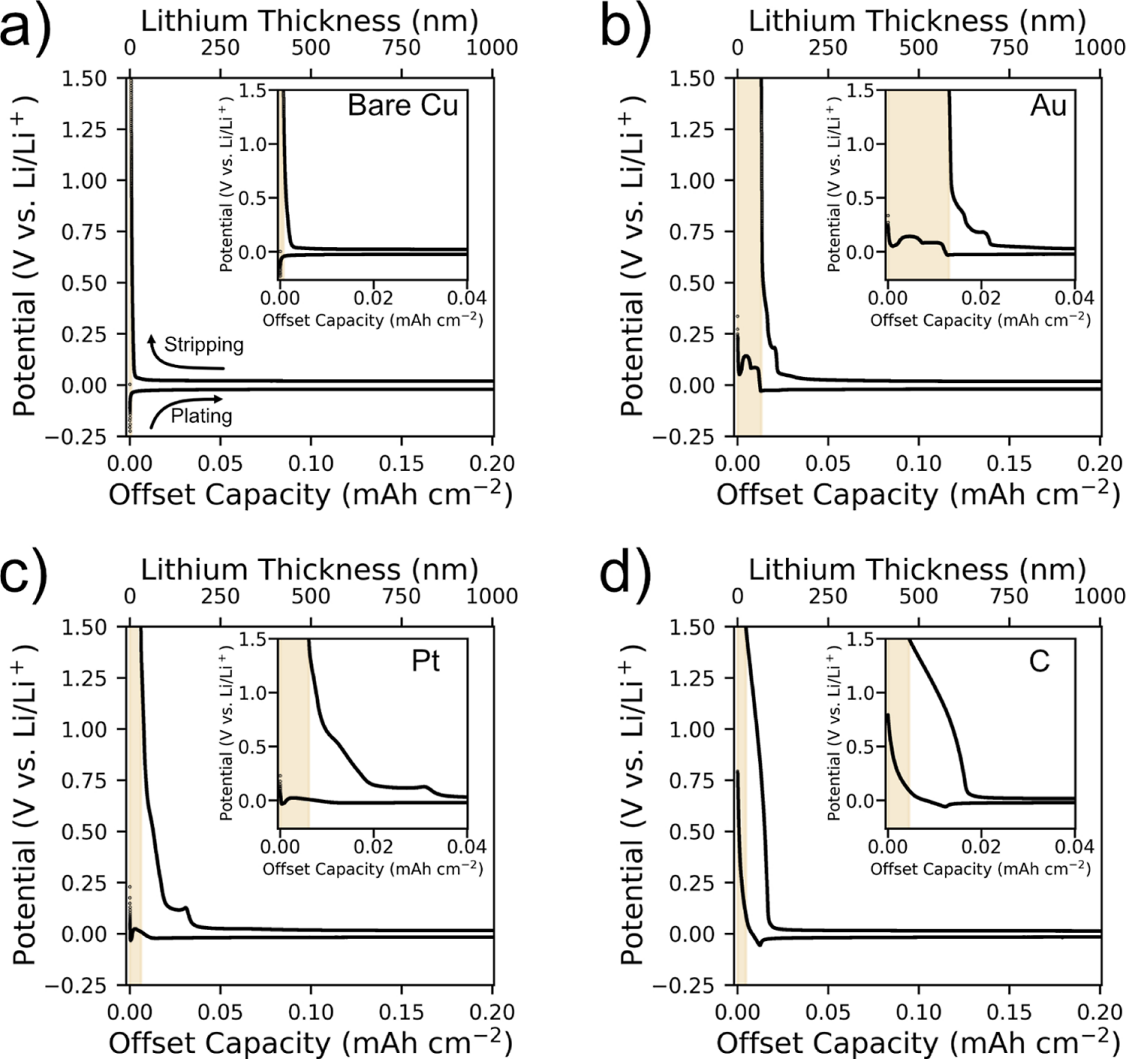
Effect of seed layers on lithium metal plating and stripping during the first cycle at a current density of 0.2 mA cm^–2^ and an offset capacity of 0.2 mA h cm^–2^. Distinct voltage profiles were observed during lithium plating and stripping for a bare copper current collector (a) and different seed layer materials of gold, platinum, and amorphous carbon (b–d). Au and Pt show alloying behavior, and C shows lithium intercalation behavior. Areas highlighted in yellow indicate lithium loss in the first cycle. The inset shows zoomed data highlighting different lithiation behaviors.

Figure 2a presents the representative voltage profiles for a bare copper CC and different seed materials (Figure 2b–d). Lithium metal of 0.2 mA h cm^–2^ was plated, corresponding to a thickness of 1 μm of dense lithium, using a current density of 0.2 mA cm^–2^. Upon application of current to the bare copper CC, the voltage exhibited a sharp decrease below 0 V vs Li/Li^+^, reaching a nucleation potential at −225 mV. This pattern, characterized by a rapid voltage drop followed by a flat voltage plateau at −20 mV, aligns with predictions from the nucleation and growth theory.^21^

Unlike copper, gold and platinum have unique interaction mechanisms with lithium. The gold layer interacts with lithium to form Li_*x*_-Au alloy phases and has a specific solubility range in lithium metal.^22^ Thus, lithium alloys with gold form a saturated phase prior to the formation of pure lithium metal. Similarly, the platinum layer, with its distinct solubility properties, provides a range of potential nucleation sites.^40^ The lithium metal plating process on gold and platinum nucleation layers is characterized by two separate potential plateaus, followed by a potential drop that signals the start of lithium plating. The plating potential for these processes reaches its minimum at approximately −30 mV. This reduced nucleation potential is attributed to the identical crystal structures of pure lithium metal (β-Li) and the solid solution surface layer, which effectively reduce nucleation barriers.^22^

Lithium plating in the presence of amorphous carbon seed layers shows a markedly different voltage profile; it shows a slower decrease in potential. This voltage decline corresponds to the initial lithiation of the carbon seed layer, indicating an intercalation behavior similar to graphite. In fact, amorphous carbon seed layers can host up to 200 mA h g^–1^ between 5 mV and 1 V vs Li/Li^+^.^41^ The drop is followed by a potential minimum at −55 mV before cells with a carbon seed layer also reach a constant voltage plateau. Despite these differences, a consistent observation at low current densities across the materials is the emergence of a flat voltage plateau at about −20 mV.

Irreversible capacity loss in the first cycle also varies between seed materials and is highlighted by the yellow areas (Figure 2). While almost no loss is observed for the bare copper CC reference, alloying materials such as gold and platinum show the greatest losses. Gold has the highest lithium loss with a peak value of 13 μA h cm^–2^, corresponding to 65 nm of dense lithium metal, while platinum has a loss of about 6 μA h cm^–2^. The greater lithium loss observed in gold during the first cycle could be attributed to the different reactivities of gold and platinum with lithium. Carbon shows the lowest irreversible lithium loss of the seed layers of about 4.5 μA h cm^–2^ in the first cycle.

**Figure 3:**
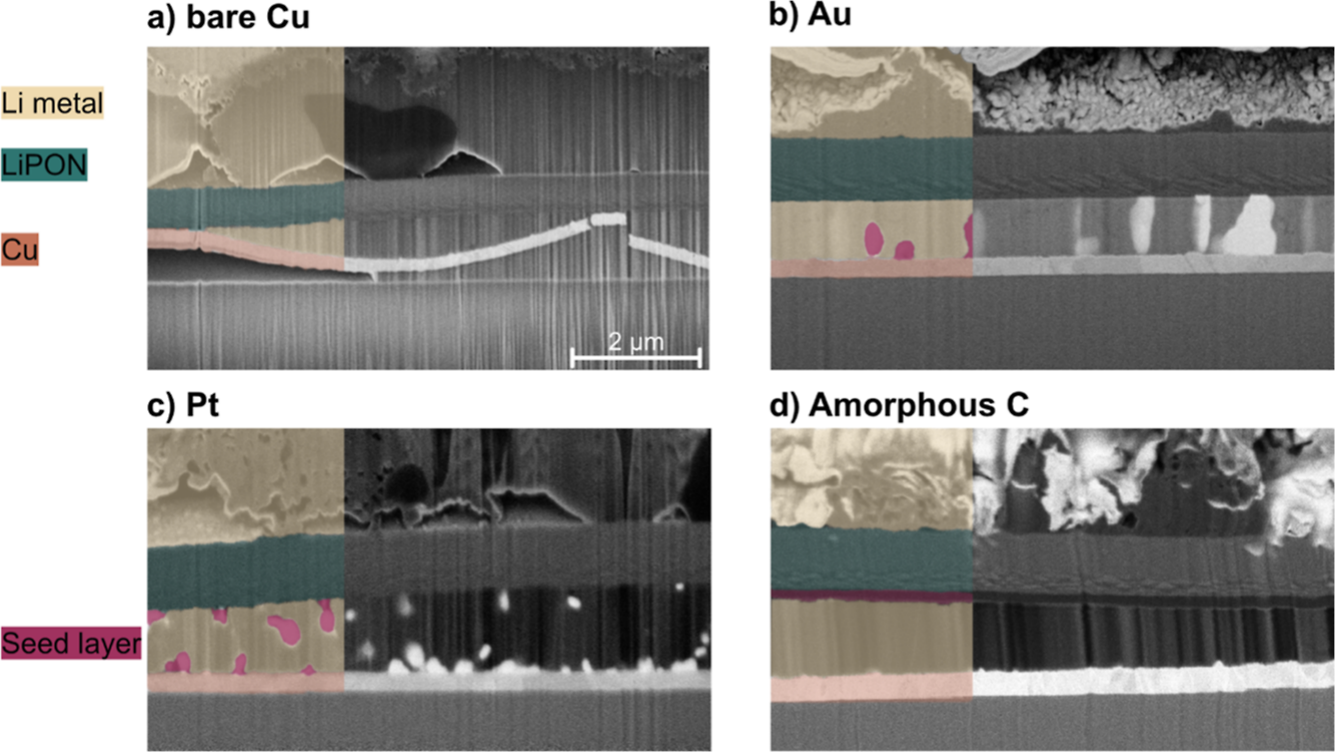
Cross-sectional SEM micrographs of the current collector-solid electrolyte interface with 0.2 mA h cm^–2^ (1 μm) plated lithium for (a) bare copper current collector and with (b–d) gold, platinum, and amorphous carbon seed layers after the first cycle. The scale bar is the same for all micrographs. All micrographs were taken in backscattered electron mode.

To better understand the plated lithium morphology, the influence of various seed layers, and irreversible lithium loss, we conducted FIB-scanning electron microscopy (SEM) analysis under cryogenic conditions. Figure 3 shows cross-sectional micrographs of the reference cell with bare copper CC and cells with gold, platinum, and amorphous carbon seed layers. Each cell has 0.2 mA h cm^–2^ of lithium metal electrochemically plated during the first cycle, which equals 1 μm of dense lithium metal.

In the copper reference cell (see Figure 3a), two large cracks are observed in the CC. These cracks can be attributed to the nonuniform deposition of lithium, which exerts mechanical forces on the copper CC, ultimately leading to its failure. This failure mechanism is a common problem in thin-film batteries, as investigated in the study of Motoyama et al.^42^ The formation of cracks in the copper CC creates energetically favorable sites for lithium nucleation. This phenomenon may also explain the observed penetration and deposition of lithium beneath the copper CC, leading to the development of gaps between the substrate and the CC. Similar cracks were detected on several other cells with bare copper CC, from both the same substrate and different batches. Additional cross-sectional SEM images of these cracks and various cells are provided in the ESI.

Figure 3b–d show cross-sectional micrographs for cells with a seed layer. The introduction of seeding layers appears to facilitate more uniform lithium deposition, which in turn reduces the mechanical stress on the CC as no cracks are observed. The gold seed layer cell contains brighter particles with sizes on the order of 1 μm within the plated lithium layer, which are likely Li–Au alloy clusters. Interestingly, the 10 nm thin gold seed layer agglomerates and forms such clusters instead of remaining in the form of a uniformly thin alloy layer. Inaoka et al.^43^ reported similar behavior at the Li/Li_3_PS_4_ interface, where the gold agglomerates into clusters. The platinum seed layer, which also forms an alloy with lithium, shows a more uniform distribution of similar but smaller clusters in the lithium metal layer. In contrast, the amorphous carbon seed layer maintains its integrity. The lithium passes through the carbon layer similarly as in our previous work^26^ and facilitates the formation of a dense and uniform lithium metal layer between the current collector and carbon interlayer.

We observed high irreversible lithium losses in the first cycle for alloying materials, especially gold and platinum, which may be related to cluster structures. Initially, gold and platinum seed layers spread uniformly over the bare copper CC (Figure 1). However, during plating, these seed layers agglomerate and form alloy clusters within the lithium layer. We speculate that only surface lithium is removed, with the remainder “trapped” inside, possibly explaining the reduced lithium loss in platinum due to its smaller area/volume ratio. In addition, carbon cells show higher irreversible capacity loss than our reference copper CC, possibly related to the formation of a Li-containing interphase (lighter contrast) seen in FIB-SEM micrographs at the lithium–carbon interface.^44^

**Figure 4:**
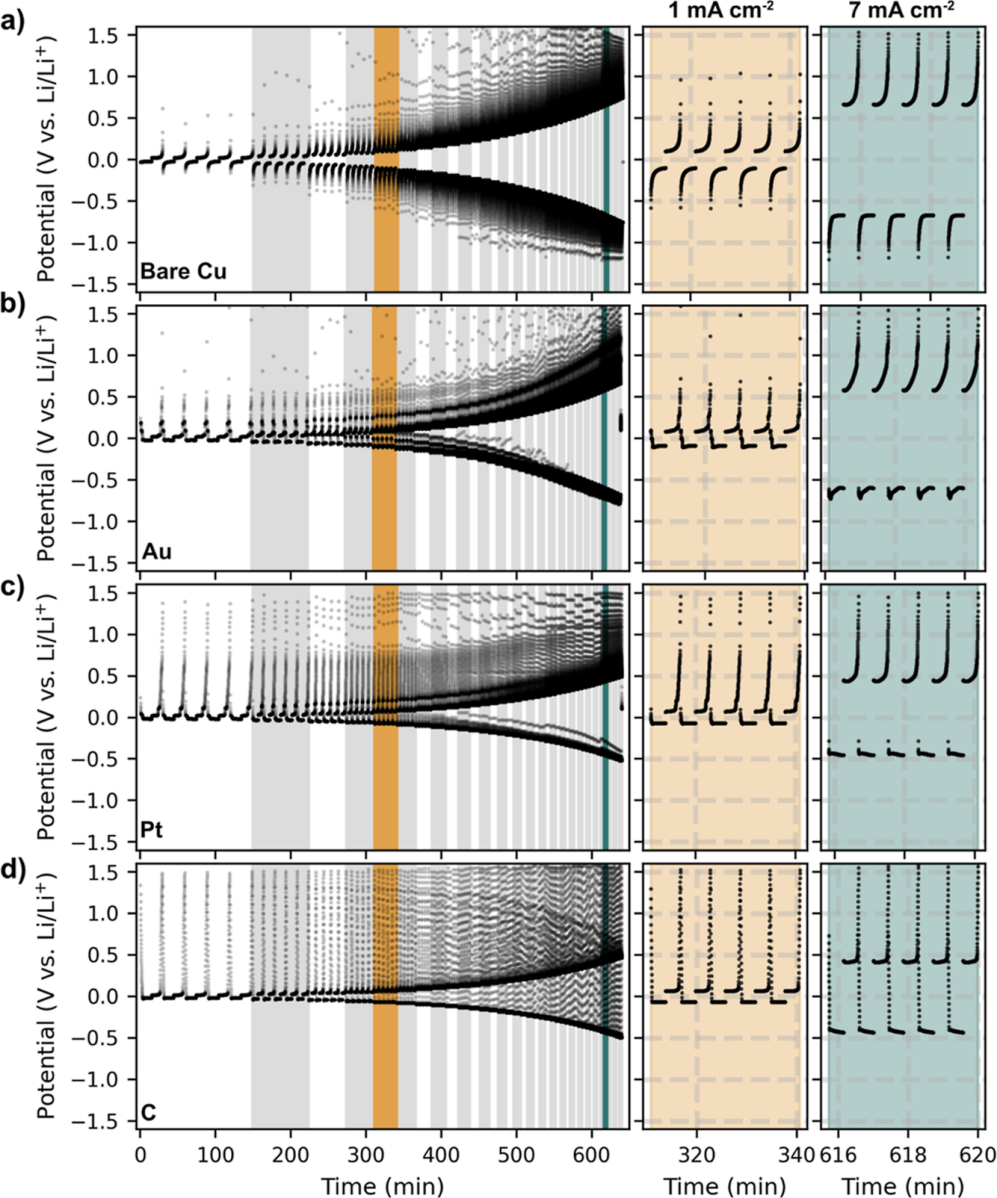
Effects of current density on lithium plating and stripping in thin-film cells for (a) bare copper CC, (b) gold, (c) platinum, and (d) amorphous carbon seed layer cells. The full data set in the first column cycled at current densities from 0.2 to 8 mA cm^–2^. The regions differentiated by varying current densities are highlighted with gray shading. Each step represents an increase of 0.2 mA cm^–2^. The second and third columns show the potential behavior at 1 mA cm^–2^ (yellow) and 7 mA cm^–2^ (green), respectively. Each density was repeated five times at an offset capacity of 0.05 mA h cm^–2^, corresponding to 250 nm of densely plated lithium.

To investigate the effect of varying current densities for lithium plating and stripping, cells were cycled at current densities ranging from 0.2 to 8 mA cm^–2^ in increments of 0.2 mA cm^–2^, as shown in Figure 4. Each current density increment was repeated five times and maintained for a time corresponding to an offset capacity of 0.05 mA h cm^–2^, equivalent to plating 250 nm of dense lithium metal.

Figure 4a shows the behavior of the reference sample with a bare CC. As the current density increases, there is a corresponding increase in potential. It is noteworthy that the half-cells do not exhibit a critical current density even at an upper limit of 8 mA cm^–2^. The critical current density is the maximum current that a cell can sustain before it shorts out. This behavior indicates inherent stability even at high current densities^45−47^ and demonstrates the robustness of the thin-film system. The voltage profiles at 1 and 7 mA cm^–2^ are shown in the second and third columns of Figure 4. In the copper reference cell at 7 mA cm^–2^, a stable plating plateau is observed at −750 mV. This plateau is consistent with the growth region identified in previous research by Pei et al.,^21^ and this stability is maintained at high current densities. In particular, the 250 nm lithium plating remains consistent, avoiding the exponential potential drops typically associated with void formation.

Figure 4b–d show the voltage profiles for cells with gold, platinum, and amorphous carbon seed layers. These cells, like the bare copper CC cells, do not reach a short circuit at the applied current density of 8 mA cm^–2^ during the plating of 250 nm dense lithium. For all seed materials tested, the lithiation plateaus are consistently observed during both the plating and stripping processes, even at higher current densities of 1 and 7 mA cm^–2^. A comparison of the bare copper CC with other seed layers reveals differences in their plating and stripping dynamics. The introduction of a thin gold seed layer improves stability, with its plateau stabilizing at −680 mV. This represents a reduction in overpotential of up to 10% at the highest current density tested, 8 mA cm^–2^. In contrast, the platinum and carbon seed layers establish their voltage plateaus at −520 and −490 mV, respectively.

**Figure 5:**
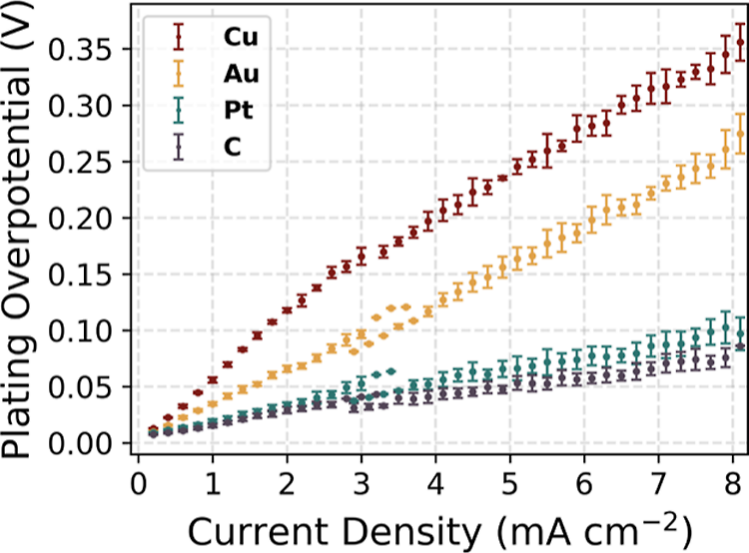
Plating overpotential response to the current density for seed layers. The potentials presented here have been adjusted to account for polarization effects due to electrolyte resistance. The error bars show the standard deviation of the data from three individual cells.

Figure 5 provides a comparison of the evolution of the plating overpotential as a function of the current density for all of the seeds. The standard deviation between individual cells per seed layer does not exceed 10%. To account for polarization effects due to electrolyte resistance, the potentials shown here have been adjusted accordingly. More detailed information on the methodology used to evaluate the plating overpotential in the growth region,^21^ including data processing and statistical analysis, can be found in the ESI Section 3.

A consistent linear trend of the increase in the plating overpotential with increasing current density is observed for all seeds. The bare copper CC cell shows the steepest increase, reaching a peak overpotential of 325 mV at a current density of 8 mA cm^–2^. The gold seed layer cell has a slightly lower rise in overpotential, reaching a maximum of 250 mV, while platinum and carbon have the lowest overpotentials of less than 100 mV at the highest current density measured.

The performance of carbon as a seed layer is characterized by a minimal increase in the plating overpotential at higher current densities, reflecting stable electrochemical plating and hence less overpotential evolution. This stability is due to the intact amorphous carbon seed layer between the current collector and the solid electrolyte—as shown in Figure 3—which ensures homogeneous plating, optimal current distribution, and minimized overpotential. It ensures uniform Li-ion diffusion, enhances surface reaction rates, inhibits lithium filament growth, and improves the reversibility of lithium plating. Our results show that carbon and lithium–platinum alloys provide better performance in lithium plating/stripping and overall battery efficiency through overpotential reduction compared with lithium–gold alloys. In addition, the promising results of two-component interlayers, namely silver/carbon^24^ and gold/carbon,^22^ confirm these findings.

## 3 Conclusions

We investigated anode-free half cells with seed layers comprising gold, platinum, or amorphous carbon placed between the LiPON solid-state electrolyte and the bare copper CC. The formation of a dense lithium metal layer between the copper CC and LiPON, which could be repeatedly plated and stripped, was demonstrated. All cells withstood current densities up to 8 mA cm^–2^ without short-circuiting, demonstrating the reliability of the thin-film configuration. Gold and platinum seed layers alloyed with lithium early in the plating process, facilitating uniform lithium metal plating on the current collector. As these layers agglomerate, they form alloy clusters distributed within the deposited lithium layer, preventing the mechanical failure of the current collector. The amorphous carbon seed layer maintains its integrity and is characterized by a uniform, dense lithium metal layer between the current collector and the seed layer. Platinum and amorphous carbon cells exhibit the lowest overpotential evolution. Amorphous carbon has been found to be a viable and cost-effective alternative to noble metals as a seed layer material.

## 4 Experimental Details

### 4.1 Fabrication

Prior to deposition, soda-lime glass substrates were thoroughly cleaned with 2-propanol. After the substrates were cleaned, a layer of copper (Cu 99.999%, Thermo Fisher Scientific) with a thickness of 250 nm was thermally evaporated onto the substrates using a Nexdep evaporator (Angstrom Engineering Inc.) at a rate of 1 Å s^–1^.

The sequence continued with the deposition of the seed layers. A 10 nm thick layer of gold (Au 99.99%, Heimerle + Meule GmbH Scheideanstalt) was thermally evaporated onto the copper current collector using the Angstrom Engineering Inc. system at a rate of 0.2 Å s^–1^. Platinum (Pt 99.99%, Plasmaterials) and amorphous carbon (99.9% pure graphite, Mo-bonded, Plansee SE) were deposited by RF magnetron sputtering using an Orion sputtering system (AJA International Inc.) at thicknesses of 10 and 50 nm, respectively. The rate for platinum was 2.5 nm min^–1^ and for carbon 0.8 nm min^–1^.

Lithium–phosphorus oxynitride (LiPON) solid-electrolyte was then RF magnetron sputtered onto the current collector/seed layer stack. This deposition was performed unheated and resulted in a 1 μm thick LiPON layer using the cosputtering technique with 2″ targets of Li_3_PO_4_ (99.95%, Kurt J Lesker Co., rate approximately 0.7 nm min^–1^) and Li_2_O (99.9%, Toshima Manufacturing, rate approximately 0.6 nm min^–1^) in a N_2_ atmosphere (flow set to 50 SCCM) at powers of 100 and 120 W, respectively, and a working pressure of 4 × 10^–3^ mbar. The target-to-substrate distance was set to 25 cm.

After the solid electrolyte was deposited, a layer of lithium (99+%, Thermo Fisher Scientific) was added by thermal evaporation at a rate of 25 Å s^–1^, forming a 6 μm thick layer (Nexdep evaporator) with 0.1 cm diameter shadow masks to evaporate individual lithium reservoirs as separate cells.

A 100 nm layer of copper was thermally deposited on top of lithium as the final step of the protocol. Throughout the deposition processes, a quartz microbalance was used to ensure the precise control of the film thicknesses.

### 4.2 Characterization

Atomic force microscopy took place in air by employing the ScanAnlyst tapping mode (Bruker Icon 3). A 2.5 μm × 2.5 μm area was scanned at a resolution of 256 × 256 pixels. Data analysis was performed with GWyddion 2.62.

Further analysis was performed using cross-sectional scanning electron microscopy. A Helios 600i TFS FIB/SEM system with a cryogenic stage was operated at −140 °C. A protective carbon layer was deposited prior to the FIB milling. The micrographs shown were taken in backscattered electron mode (2 kV and 0.69 nA).

The electrochemical characterization process was performed under an Ar atmosphere at room temperature with a Squidstat potentiostat (Admiral Instruments). A detailed protocol can be seen in Supporting Information Section 5.

## Supporting Information Available

Table summarizing AFM parameters; influence of 100 nm Au seed layer on Li plating/stripping; description of overpotential calculation methodology; cross-sectional SEM images of cracks in bare copper layers; interphase formation at the carbon seed layer; and detailed cycling procedure used for plating and stripping (PDF)
