# Supplementary material for: Influence of Au, Pt, and C Seed Layers on Lithium Nucleation Dynamics for Anode-Free Solid-State Batteries
Source: ACS Appl Mater Interfaces. 2023 Dec 21;16(1):695–703. doi: 10.1021/acsami.3c14693 (PMC10788862; doi:10.1021/acsami.3c14693)
Supplement: Supplementary file 1 — am3c14693_si_001.pdf [file am3c14693_si_001.pdf]

# Supporting Information: Influence of Au, Pt, and C Seed Layers on Lithium Nucleation Dynamics For Anode-Free Solid-State Batteries

André Müller,* Luis Paravicini, Jędrzej Morzy, Maximilian Krause, Joel Casella, Nicolas Osenciat, Moritz H. Futscher, and Yaroslav E. Romanyuk*

*Laboratory for Thin Films and Photovoltaics, Empa - Swiss Federal Laboratories for Materials Science and Technology, Überlandstrasse 129, CH-8600 Dübendorf, Switzerland*

E-mail: andre.mueller@empa.ch; yaroslav.romanyuk@empa.ch

Phone: +41 58 765 4608; +41 58 765 4169

# 1 AFM characterization

Table S1: Summary of AFM-derived surface parameters for the investigated materials

| | | **Au** | **C** | **Cu** | **Pt** |
|---|---|---|---|---|---|
| Average value | nm | 9,87 | 7,53 | 8,29 | 9,05 |
| RMS roughness (Sq) | **nm** | **2,16** | **2,15** | **2,45** | **2,18** |
| RMS (grain-wise) | nm | 2,16 | 2,15 | 2,45 | 2,18 |
| Mean roughness (Sa) | nm | 1,68 | 1,70 | 1,95 | 1,75 |
| Skew (Ssk) | | 0,36 | 0,28 | 0,18 | -23,31 |
| Excess kurtosis | | 1,41 | 0,38 | 0,51 | -0,18 |
| | | | | | |
| Minimum | nm | 0 | 0 | 0 | 0 |
| Maximum | nm | 21,1 | 16,6 | 21,54 | 15,68 |
| Median | nm | 9,84 | 7,47 | 8,29 | 9,06 |
| Maximum peak height (Sp) | nm | 11,23 | 9,07 | 13,25 | 6,63 |
| Maximum pit depth (Sv) | nm | 9,87 | 7,53 | 8,29 | 9,05 |
| Maximum height (Sz) | **nm** | **21,11** | **16,60** | **21,54** | **15,68** |
| | | | | | |
| Projected area | **$\mathbf{nm^2}$** | **250000** | **250000** | **250000** | **250000** |
| Surface area | $nm^2$ | 270238 | 262507 | 273273 | 266517 |
| Surface slope (Sdq) | | 0,45 | 0,34 | 0,47 | 0,39 |
| Volume | $nm^3$ | 2468253 | 1881908 | 2071644 | 2262142 |
| Variation | $nm^2$ | 89922,3 | 72074,8 | 95299,1 | 81495,4 |
| Inclination $\theta$ | deg | 0,32 | 0,07 | 0,08 | 0,23 |
| Inclination $\varphi$ | deg | -170,59 | -157,96 | -148,21 | -160,88 |
| | | | | | |
| Scan line discrepancy | | 0,04 | 0,03 | 0,04 | 27,88 |

# 2 Impact of seed layer thickness on Li plating/stripping

Figure S1 illustrates the dynamics of Li metal plating and stripping for a 100 nm Au seed layer during its first cycle. Notably, a pronounced 50% loss of the initial Li deposition is evident, though the (de)lithiation plateaus are retained.

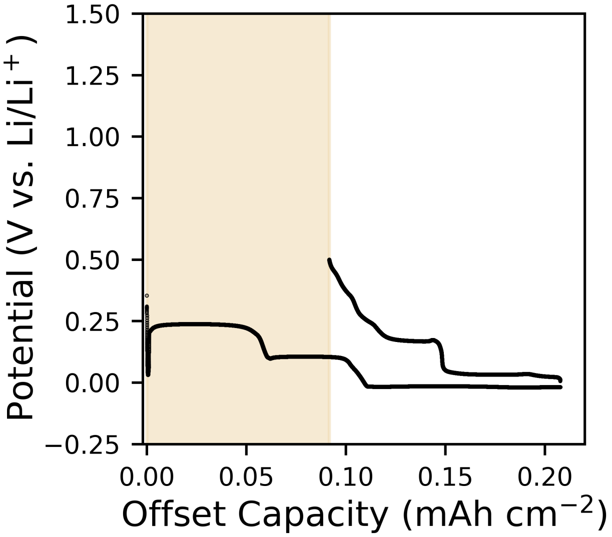


Figure S1: Effect on Li metal plating and stripping during first cycle at a current density of 0.2 mA $^{-2}$ and a capacity of 0.2 mAh $^{-2}$ for 100 nm Au seed layer.

In Figure S2 a current density evaluation of the same 100 nm Au seed layer is shown. Persistent Li losses are observed, culminating in an early short-circuit at approximately 4 mA cm$^{-2}$ - a threshold that is significantly lower than that observed for 10 nm layers. The data highlight the significant loss in performance associated with increasing Au seed layer thickness.

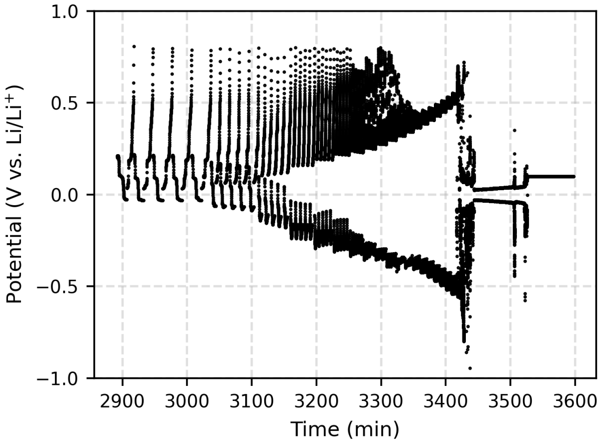


Figure S2: Effects of current density on Li plating and stripping in thin-film cells with 100 nm Au seed layer.

# 3 Methodolgy: calculation of overpotential statistics

The focus of this study is to determine the overpotential development in the growth region of various materials. The methodology for determining the overpotential for the tested seed layer materials and the reference is described below.

Initial electrochemical tests were performed on these materials as described in the manuscript. For each material, the overpotential was determined by evaluating the average potential in the growth region[21], taking into account ohmic losses. The plating potential was determined by averaging the potentials in the growth region, followed by an adjustment for ohmic losses - calculated from the product of current density and system impedance.

The ohmic losses, which are primarily due to the electrical resistance of the solid electrolyte, were adjusted using an impedance-based correction factor applied to the calculated potential of each material. Importantly, all cell electrolytes were sputtered in the same run to ensure consistency.

Mathematically, the adjusted plating potential can be expressed as

$$V_{plating} = |\overline{V_{plateau}}| - Z_{real} A j \tag{1}$$

Where $|V_{plateau}|$ [V] symbolizes the averaged potential within the growth region, $Z_{real}$ [$\Omega$] is the impedance of the system, $A$ [$cm^2$] is the surface area, and $j$ [$Acm^{-2}$] refers to the current density.

The nucleation region is where new lithium metal particles begin to form on the electrode. It begins with small clusters of lithium atoms coming together and, once stable, growing into larger particles. The growth region is where these lithium metal particles expand in size.[21] Several factors influence these processes, such as voltage differences, current density, and electrode structure. The goal is uniform, compact lithium deposition to prevent dendrite formation, which can short-circuit the battery. The region is dynamic and changes during charging and discharging due to the continuous plating and stripping of lithium metal.

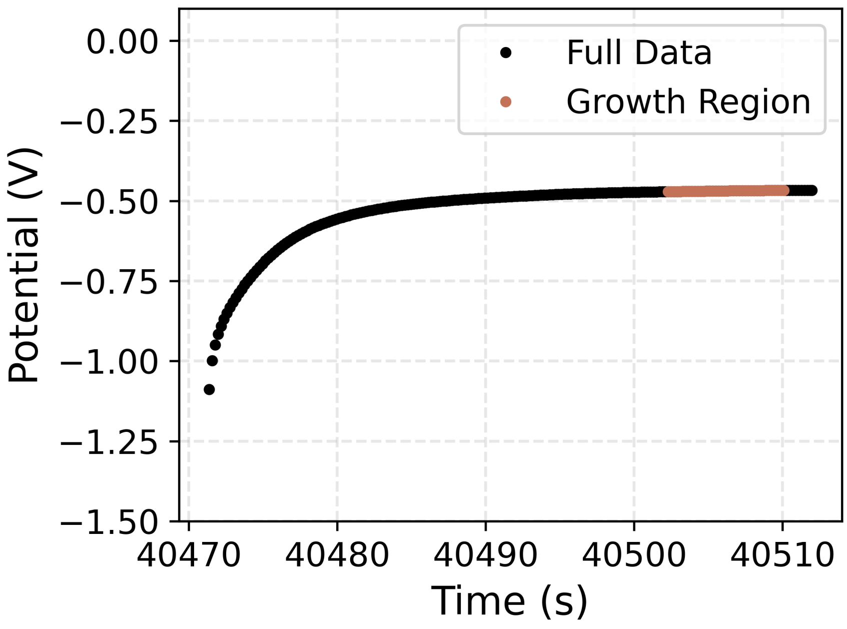


Figure S3: Plot of potential versus elapsed time. The entire data set is represented by black dots, while the specific subset representing the growth region of the data is highlighted by colored dots.

# 4 Cracking of bare copper CC

During the process of plating metal lithium onto the bare copper CC, it was observed that the copper layer began to exhibit cracks, as shown in Figure S4. These cracks provided a pathway for the lithium metal not only to plate beneath the copper layer but also to diffuse into the soda-lime glass substrate, as depicted in Fig. 3a. The origin of these cracks can be traced back to the mechanical stress exerted on the copper CC films during the plating process.[41] This stress led to the deformation of the copper, resulting in the aforementioned cracks.

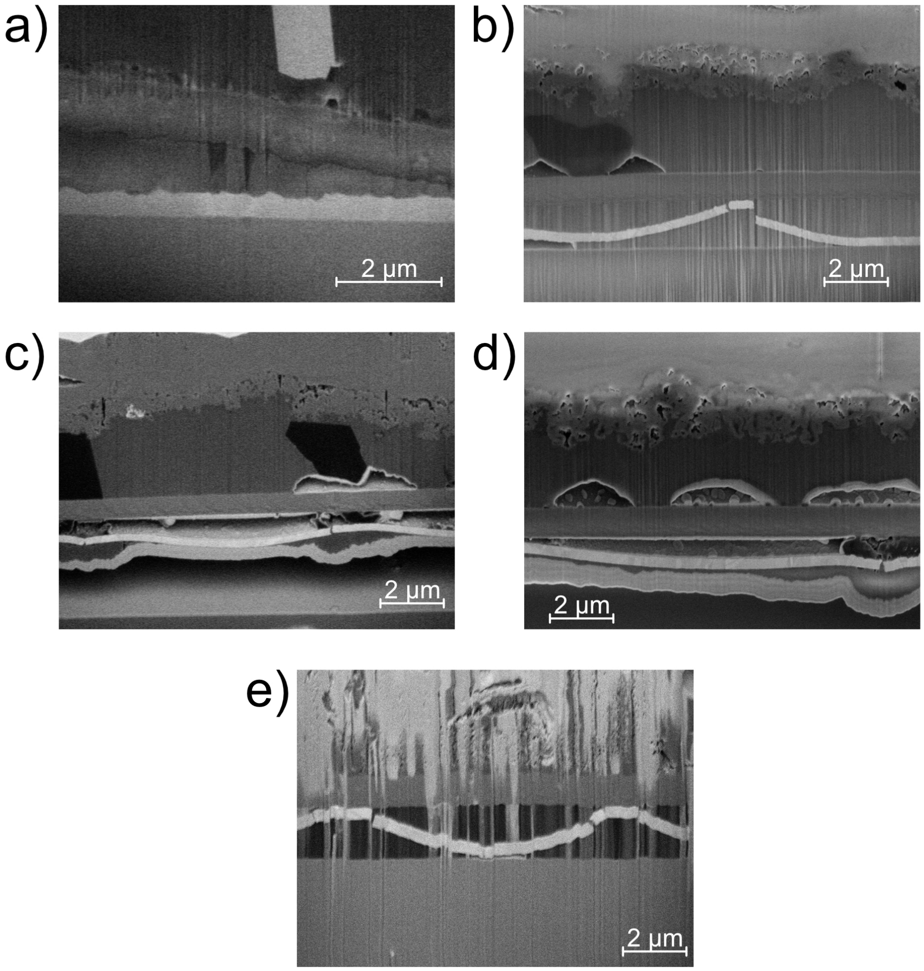


Figure S4: Cross-sectional micrographs of the reference architecture Cu/LiPON/Li/Cu with 0.2 mAh cm$^{-2}$ (1 µm) of lithium plated. a) shows non-uniform plating, while b)-e) show cracks in the copper CC.

# 5 Interphase formation carbon seed layer

Figure S5 provides a magnified view of the interface where lithium has been deposited. There appears to be a distinct brighter region, as can be seen more clearly in the magnified view. In our study, we observed a higher irreversible capacity loss in the carbon cells compared to the bare copper CC reference cell. This brighter region is likely indicative of interphase formation.

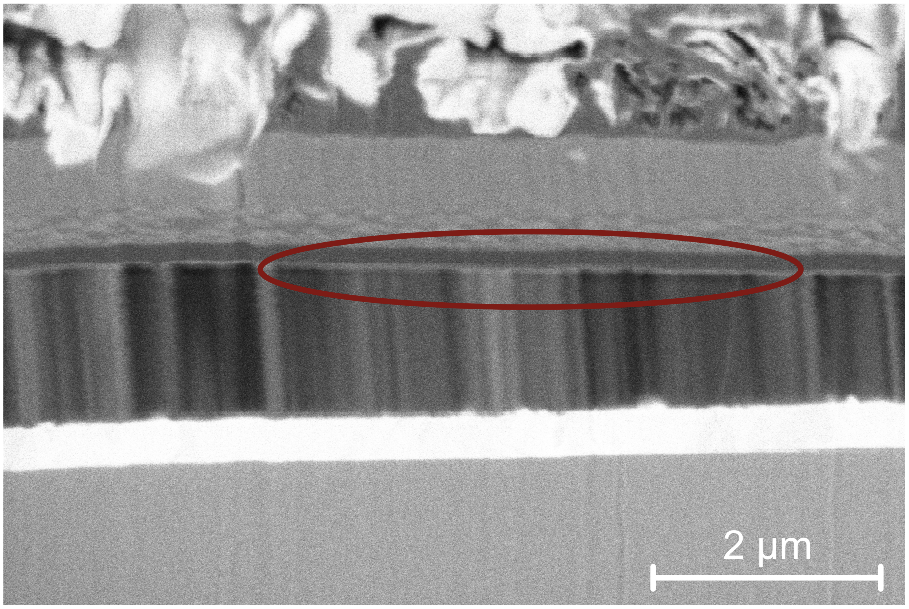


Figure S5: Cross-sectional FIB-SEM micrographs depicting the interface between the plated lithium and the carbon layer in magnified view.

## 6 Detailed cycling procedure

The plating/stripping cycling protocol is outlined below and shown schematically in Figure S6.

1. Initially, a constant current plating of 0.2 mAh $cm^{-2}$ was carried out, utilizing a current density of 0.2 mA $cm^{-2}$.

2. Following this, the plated lithium was stripped using the same current density, continuing until a cut-off potential of 1.5 V was attained.

3. To ensure the complete removal of residual lithium, we employed constant voltage stripping.

4. Subsequently, we carried out repeated cycles of constant current plating and stripping:

- Each plating and stripping cycle was executed five times consecutively.

- After completing these five cycles, the current density was incrementally increased by 0.2 mA $cm^{-2}$. This step-by-step increment was continued (i.e., 0.2, 0.4, 0.6, 0.8 mA $cm^{-2}$ and so on) until a current density of 8 mA $cm^{-2}$ was reached.

During the repeated cycles (as mentioned in step 4), we did not utilize constant voltage stripping, but the cut-off potential of 1.5 V was consistently maintained.

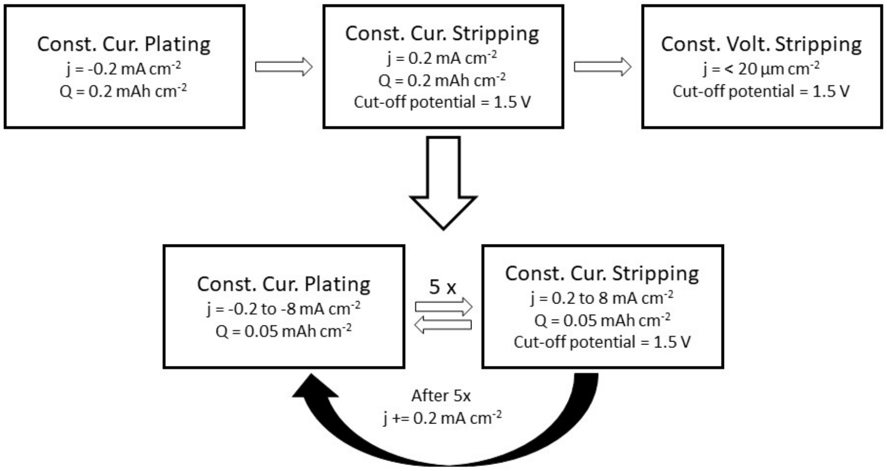


Figure S6: Detailed cycling procedure for the plating/stripping experiment.
